# Large scale RNAi screen in *Tribolium* reveals novel target genes for pest control and the proteasome as prime target

**DOI:** 10.1186/s12864-015-1880-y

**Published:** 2015-09-03

**Authors:** Julia Ulrich, Van Anh Dao, Upalparna Majumdar, Christian Schmitt-Engel, Jonas Schwirz, Dorothea Schultheis, Nadi Ströhlein, Nicole Troelenberg, Daniela Grossmann, Tobias Richter, Jürgen Dönitz, Lizzy Gerischer, Gérard Leboulle, Andreas Vilcinskas, Mario Stanke, Gregor Bucher

**Affiliations:** Johann-Friedrich-Blumenbach-Institut, GZMB, Georg-August-Universität Göttingen, Justus-von-Liebig-Weg 11, 37077 Göttingen, Germany; Department Biologie, Entwicklungsbiologie, Friedrich-Alexander-Universität Erlangen-Nürnberg, Staudtstraße 5, 91058 Erlangen, Germany; Institut für Entwicklungsbiologie, Universität zu Köln, Zülpicher Straße 47b, 50674 Cologne, Germany; Institut für Mathematik und Informatik, Ernst Moritz Arndt Universität Greifswald, Walther-Rathenau-Straße 47, 17487 Greifswald, Germany; Institut für Biologie, Neurobiologie, Freie Universität Berlin, Königin-Luise-Str. 28/30, 14195 Berlin, Germany; Institut für Phytopathologie und Angewandte Zoologie, Justus-Liebig-Universität Gießen, Heinrich-Buff-Ring 26-32, D-35392 Gießen, Germany

**Keywords:** Pest control, RNAi, RNAi target gene, iBeetle, Tribolium, Proteasome

## Abstract

**Background:**

Insect pest control is challenged by insecticide resistance and negative impact on ecology and health. One promising pest specific alternative is the generation of transgenic plants, which express double stranded RNAs targeting essential genes of a pest species. Upon feeding, the dsRNA induces gene silencing in the pest resulting in its death. However, the identification of efficient RNAi target genes remains a major challenge as genomic tools and breeding capacity is limited in most pest insects impeding whole-animal-high-throughput-screening.

**Results:**

We use the red flour beetle *Tribolium castaneum* as a screening platform in order to identify the most efficient RNAi target genes. From about 5,000 randomly screened genes of the iBeetle RNAi screen we identify 11 novel and highly efficient RNAi targets. Our data allowed us to determine GO term combinations that are predictive for efficient RNAi target genes with proteasomal genes being most predictive. Finally, we show that RNAi target genes do not appear to act synergistically and that protein sequence conservation does not correlate with the number of potential off target sites.

**Conclusions:**

Our results will aid the identification of RNAi target genes in many pest species by providing a manageable number of excellent candidate genes to be tested and the proteasome as prime target. Further, the identified GO term combinations will help to identify efficient target genes from organ specific transcriptomes. Our off target analysis is relevant for the sequence selection used in transgenic plants.

**Electronic supplementary material:**

The online version of this article (doi:10.1186/s12864-015-1880-y) contains supplementary material, which is available to authorized users.

## Background

Alternative strategies for sustainable agriculture attract increasing attention because cultural practices and agrochemicals are not sufficient or permanently successful in keeping plant pathogens and pest insects under control. One of the most challenging problems in modern plant protections is the rapid adaptation of pest insects to insecticides which imposes both increasing costs of chemical plant protection and growing public concern about the hazards on environment and human health [1–3]. Transgenic plants expressing *Bacillus thuringiensis* (Bt) toxins provide an alternative option to engineer insect-resistant crops. Despite the still lasting public debate about the introduction of genetically modified plants into the field, the production of transgenic plants increases globally. However, economic important pest insects such as the western corn rootworm, *Diabrothica virgifera virgifera*, evolved resistance against Bt-toxins expressed in engineered maize [4]. The questionable sustainability of this “first generation transgenic crops” and the fact that Bt-toxins can also impair non-target insects raised a demand for alternative and more pest specific transgenic approaches in plant protection among which the RNA-Interference (RNAi) appears most promising [5, 6]. Targeted silencing of gene expression by injection of corresponding double-stranded RNA (dsRNA) has become a major tool in functional analysis of genes in insects [7–10]. In many insects, like the red flour beetle *Tribolium castaneum*, the RNAi effect spreads throughout the animal and is even transmitted to the offspring [11–14]. Importantly, some insects elicit the RNAi response upon ingestion of dsRNA. Based on this, dsRNAs have been implicated as a new generation of species-specific insecticides that kill insects upon oral up-take [15, 16]. Consequently, the possibility to engineer crops expressing double-stranded RNA which can silence target genes in pest insects after ingestion provides the opportunity for the development of species-specific control measures. One current limitation is the identification of dsRNAs that kill the targeted pest insect even when provided in minute amounts. First, the production of dsRNA in transgenic crops is limited and second, only small amounts are delivered to the pest insect via the ingested plant food. Unfortunately, the systematic identification of suitable RNAi target genes is challenging in pest species because they are usually difficult to rear in the lab and often lack the required genomic and genetic tools for a whole-animal-high-throughput-screen. The main insect model system *Drosophila melanogaster* lacks systemic RNAi and is therefore not well suited to screen for RNAi target genes [17].

The red flour beetle *Tribolium castaneum* has developed to an excellent insect model organism. It can be reared in large amounts in the lab, reproduces all year round, was the first beetle to be sequenced and both genetic and transgenic tools are available [18–22]. Its main strength is the strong and systemic RNAi response [10–12], which allowed performing a large scale unbiased RNAi screen (iBeetle Screen) [23, 24 ].

The currently prevailing small scale approaches of RNAi target identification may have missed the most efficient RNAi target genes. We reasoned that the red flour beetle would be an appropriate screening platform for the independent large scale identification of the most efficient RNAi target genes in insects. The respective orthologs could then be tested as RNAi target genes in other pest species. Therefore, we mined approximately 5.000 experiments of the iBeetle screen in order to identify those treatments that induced death of the injected animals most rapidly. We tested selected dsRNAs further by titration experiments and found that a number of them performed better than previously used RNAi target genes. Hence, the orthologs of these genes are novel prime candidates for RNAi based approaches of pest control of other pest species. Based on this set of efficient RNAi target genes, we identified GO term combinations that are predictive of good RNAi target genes and which identify the proteasome as a prime target. Finally, we tested and refuted the hypothesis that the efficiency of RNAi mediated pest control could be enhanced by synergistic action in double knock-downs. Further, we find that potential off-target-sequences occur independently of protein sequence conservation. Hence, off target effects can hardly be ruled out completely and efforts to reduce ecological side effects need to focus on selected species to be protected.

## Results and discussion

### Large scale RNAi screen identifies novel RNAi target genes

**Fig. 1:**
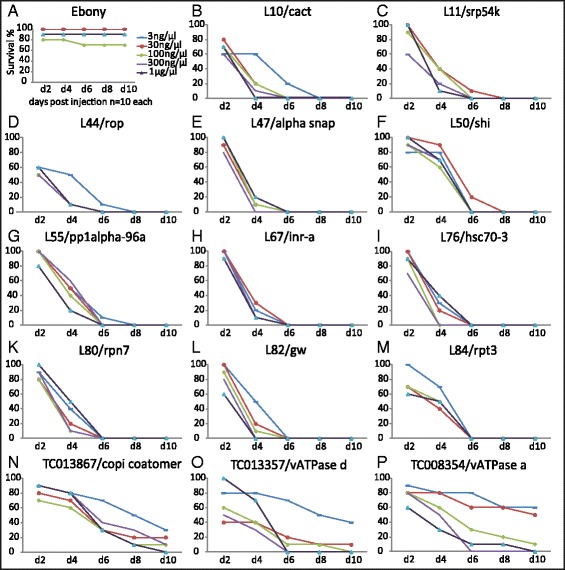
Identification of novel RNAi target genes in *Tribolium castaneum.* The survival after knock-down of the 11 most efficient RNAi target genes identified in our screen is shown. dsRNAs of different concentrations were injected into 10 *Tribolium* larvae, respectively, and the survival rate was recorded every second day. **a** The pigmentation gene *Tc-ebony* was used as negative control. **b**–**m** Most of our novel RNAi target genes showed a larval mortality of 100 % on day eight and 80 % on day six post treatment for every dsRNA concentration. **n**–**p** RNAi treatment of commonly used targets based on the seminal paper of Baum et al. [15] revealed a lower efficiency (see also Additional file 2: Fig. S1B)

So far, the targets for dsRNA based pest control have been identified by small scale screens and on knowledge based approaches, i.e. by testing genes where previous data indicated an essential function. However, this approach will miss many genes that have not yet been linked to an essential function in one of the model species. Therefore, we screened the data produced by the large scale RNAi screen *iBeetle* (Bucher, Klingler, personal communication), where randomly selected genes were downregulated by injection into pupae and larvae and the resulting phenotypes were documented in the iBeetle-Base [23]. In the iBeetle screen, about 5,000 genes had been screened [24]. Of those, 100 revealed ≥90 % mortality both 9 days after pupal and eleven days after larval dsRNA injection (Additional file 1: Table S1). In order to confirm these results and to test for sensitivity, we injected different concentrations (3 ng/μl, 30 ng/μl, 100 ng/μl, 300 ng/μl and 1 μg/μl) of the same dsRNAs into 10 larvae, respectively, and scored the survival rate every second day. The most effective 40 genes caused a mortality of 50–100 % at day eight post injection using the lowest concentrated dsRNA (Additional file 1: Table S2, Additional file 2: Figure S1 A). We focused on the 11 most effective target genes, which were marked by mortality of 100 % on day eight and at least 80 % on day six post injection (Fig. 1b–m). This high degree of lethality was confirmed by repeating the experiment using non-overlapping dsRNA fragments (1 μg/μl) making off target effects improbable (Additional file 1: Table S3).

For comparison, we performed the same experiment with the orthologs of five RNAi target genes published in the seminal paper of Baum et al. [15], which caused lethality in the western corn rootworm (WCR) upon dsRNA ingestion. Indeed, the *Tribolium* orthologs of these genes induced a high degree of mortality, but especially with low dsRNA concentrations, the mortality did not reach the one of the 11 candidates identified in this study (Fig. 1n–p, Additional file 2: Figure S1 B).

In order to check for stage dependence of the lethal effect, we injected dsRNAs targeting these 11 genes into ten adult beetles, respectively. Mortality rate on day eight post treatment was at least 90 % with 100 ng/μl dsRNA concentration while injection of 3 ng/μl dsRNA led to lower degree of mortality indicating a concentration response curve at these concentrations (Additional file 2: Figure S2). In summary, we identified 11 novel RNAi target genes that efficiently and rapidly induce lethality at larval, pupal and adult stages even at low doses of dsRNA and are more efficient than previously used target genes at least in *Tribolium*.

### Double RNAi led to additive but not to synergistic effects

**Fig. 2:**
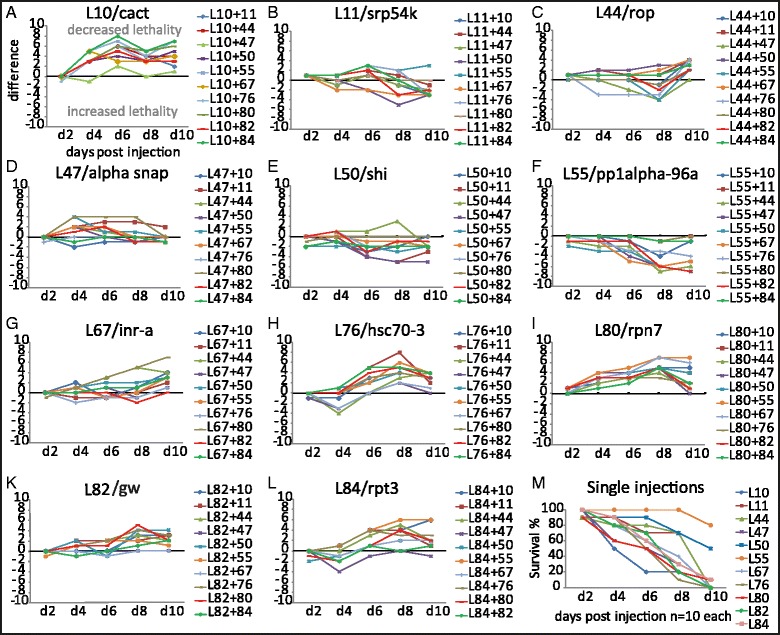
Double RNAi led to additive but not synergistic effects. **a**–**l** Double RNAi treatments were performed and the number of surviving animals at different days post injection was documented (see values in Additional file 1: Table S4). From these values, the number of surviving animals of the respective single treatments (**m**) was subtracted, such that higher lethality in the double treatment is indicated by negative values in the panels. For every target gene, ten different pairwise combinations were injected with a total dsRNA concentration of 0.5 ng/μl and compared to the single injections (0.5 ng/ul total concentration). **m** Single injection of the 11 RNAi target genes were performed with a dsRNA concentration of 0.5 ng/μl

We asked, whether the lethality of RNAi treatments can be increased synergistically by combined injection of two dsRNAs targeting different essential genes. All 55 pairwise combinations of the 11 top RNAi target genes were injected into larvae at the same end concentration as the single injections (i.e. either 0.5 ng/μl of one dsRNA or 0.25 ng/μl of two dsRNAs summing up to 0.5 ng/μl end concentration) and the survival was documented (Additional file 1: Table S4). We find no indication for synergism. Instead, the observed deviations from the baseline in some combinations are explained by additive effects: The most efficient targets become less penetrant when “diluted” with less effective dsRNAs (e.g. Fig. 2a) while less efficient targets become more potent when supplemented with stronger target genes (e.g. Fig. 2f). In conclusion, we found no indication for synergistic effects that would be able to significantly enhance the technique (Fig. 2).

### Degree of sequence conservation does not strongly influence the number of off-targets

**Fig. 3:**
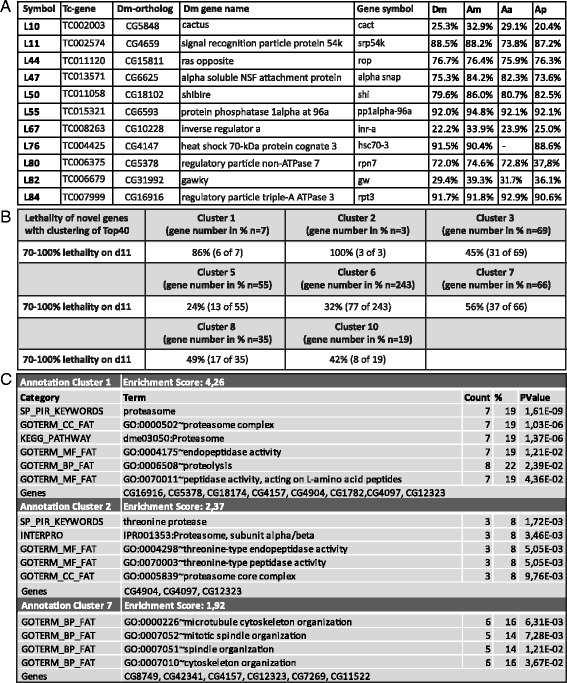
GO term clustering reveals the proteasome as efficient target. **a** All 11 top RNAi target genes have *Drosophila* orthologs and overall, their protein sequence identity with other species is high. Only Cact, Inr-a and Gw show a low degree of sequence conservation. Dm *Drosophila melanogaster*, Am *Apis mellifera*, Aa *Aedes aegypti,* Ap *Acyrthosiphon pisum*. **b** GO term clustering of the top 40 RNAi target genes revealed ten clusters with enriched biological processes. Searching for genes that share the respective GO term combinations identified additional RNAi target genes. For cluster 1, seven novel genes with the respective combination were found and six of them (86 %) turned out to be highly lethal at day 11 (d11) after injection. Cluster 1, 2 and 7 showed the highest predictive power. **c** The GO terms of the most predictive clusters are shown. *Count* is the number of genes annotated with a given term. GOTERM_BP_FAT are annotations with respect to biological processes (see Additional file 2: Figure S4 for all clusters)

In order to protect non-target organisms it would be desirable to use dsRNA fragments that are specific to the pest species and do not contain sequences targeting genes in non-target organisms (off targets). Therefore, we asked whether protein sequence conservation of our RNAi target genes correlated with the number of potential off target sites in other species. On the protein sequence level most of our RNAi target genes showed a strong conservation between some well sequenced species covering insect diversity (*Drosophila melanogaster, Aedes aegypti* (Diptera), *Apis mellifera* (Hymenoptera), *Acyrthosiphon pisum (*Hemiptera). L10, L67 and L82 were the least conserved (Fig. 3a). For protein L76 no ortholog could be identified in *Aedes aegypti* (Fig. 3a; see Additional file 2: Figure S3 H for phylogenetic analysis).

**Fig. 4:**
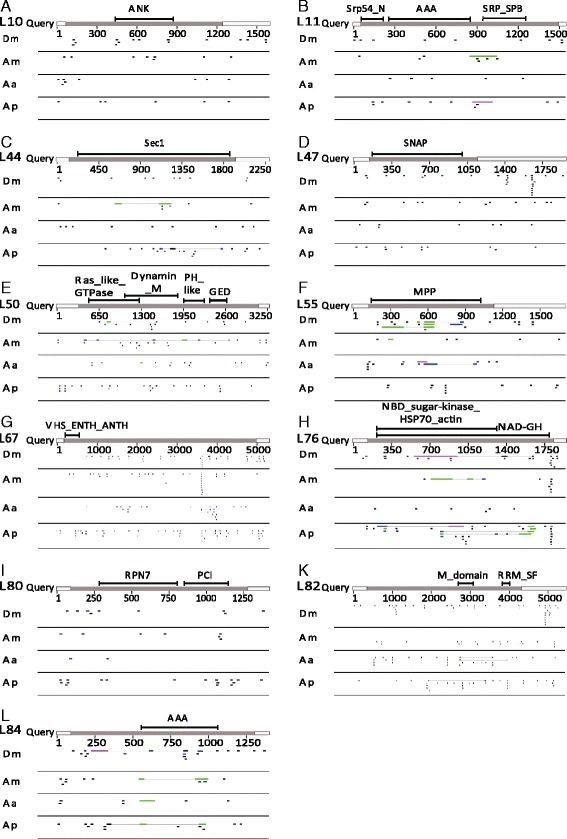
Location of potential off target regions is not restricted to stretches of conserved protein sequence. **a-l** The non-coding and rapidly evolving UTRs are indicated as *open bars*, the coding sequence as *grey bar*. Conserved protein domains are indicated above the coding sequence by *black bars* and the protein domain name. Sites with ≥17 nt identity were identified. The hits were plotted at the respective position of the query for each species (rows below the query). We did not find strong if any correlation between the degree of sequence conservation (UTR < coding sequence < conserved domains) and the location of potential off target sites. This indicates that it will be difficult if not impossible to exclude off target effects when misexpressing dsRNAs in plants. Multiple isoforms of different genes were not figured. Dm *Drosophila melanogaster*, Am *Apis mellifera*, Aa *Aedes aegypti*, Ap *Acyrthosiphon pisum*

DsRNAs are processed by the enzyme Dicer into 21–23 nt long short interfering RNAs (siRNAs). After incorporation into the RNA-induced silencing complex (RISC), they serve as template to recognize the complementary mRNA and target it for destruction [9]. However, siRNAs with an exact sequence identity of ≥17 nt can already induce off target effects [25]. Therefore, we searched the nucleotide sequences of the 11 *Tribolium* target genes against the well annotated NCBI transcriptome databases of the abovementioned species for ≥17 bp long stretches of identity (http://blast.ncbi.nlm.nih.gov/Blast.cgi) [26]. For visualization, we plotted these putative off target regions targeting in genes of other species against the RNAi target gene nucleotide sequence (Fig. 4).

We did not find an overt correlation between the conservation of a protein and the number of potential off target regions (compare the most diverged genes L10, L67 and L82 with more conserved genes in Fig. 4). Likewise, within a given gene we found no enrichment of potential off target regions in more conserved stretches of the sequence (e.g. conserved protein domains) compared to less conserved stretches (e.g. non-coding UTRs; Fig. 4). Importantly, the location of potential off target sites was generally different for the different species. Together, these observations indicate that the number and location of the off target sites does not strongly correlate with protein sequence conservation. Further, given the vast diversity of taxa even in small habitats, it will be difficult if not impossible to identify target sequences without potential off-target site in any other species.

Note that most of the identified potential 17 bp off-target-regions will not lead to any RNAi response and that at least half of the potentially targeted genes will not lead to lethality [24]. Further, only individuals that actively eat the protected plants will suffer. Hence, the species specificity of the RNAi based technique remains unchallenged when compared to other methods that usually target all or at least many species. Nevertheless, efforts to increase safety should focus on selected species (like bees) that need to be protected in the respective given ecological setting.

### GO term cluster identifies the proteasome as prime RNAi target gene

We tried to identify common properties of our identified RNAi targets, because this information might help identifying novel RNAi targets in species less amenable to large scale screens. We first analyzed the adult body expression levels in *Tribolium* and compared them to their *Drosophila* orthologs [27]. A striking pattern was not found apart from generally high expression of the *Drosophila* orthologs, which was specifically true for the central nervous system (Additional file 2: Figure S3 M).

Next, we searched for GO term clusters of the top 11 and top 40 RNAi targets [28, 29] using *Drosophila melanogaster* GO term annotations as the background. The clustering of the 40 RNAi target genes resulted in 10 clusters (Additional file 2: Figure S4). In order to test, in how far these GO terms are predictive, we identified 1328 *Drosophila* genes sharing the same GO term combinations of at least one of these clusters. For those, we determined the respective *Tribolium* orthologs and found that 502 of them had by chance been included in the iBeetle screen. Almost all novel genes identified by GO term combinations of cluster 1 and 2 (GO terms related to proteasome function) showed a strong lethality in the iBeetle screen (≥70 % after pupal or larval injection; Fig. 3b, c). Cluster 7 (GO terms related to cytoskeleton organization) represented the third-best cluster with 37 of 66 novel genes showing lethality in the screen (Fig. 3b, c). The clustering of the top 11 RNAi target genes did not result in clusters, which were able to predict novel RNAi target genes, which could be due to the low number of input genes, which makes statistical analysis challenging (not shown). Taken together, this analysis reveals GO term combinations that are predictive for potential RNAi target genes and reveal the proteasome as prime target for RNAi based insecticides.

## Conclusion

We extend the number of potential RNAi target genes by two means: First, we used the red flour beetle *T. castaneum* as screening platform in order to identify RNAi target genes by the hypothesis independent large scale RNAi screen iBeetle [24]. Indeed, novel RNAi target genes were revealed that efficiently induced death in the pest species *Tribolium castaneum* and that performed better compared to previously used genes. Hence, the RNAi target genes identified in this study are excellent candidates in future efforts in other pest species. Secondly, we identify GO term clusters that are predictive for genes with lethal phenotype upon RNAi. The search for the most lethal genes in e.g. gut transcriptomes of pest species will be accelerated by selecting genes that show these GO term combinations. Third, the proteasome appears to be a prime target for pest control. At this stage we cannot exclude that oral intake of dsRNA will have different dynamics than dsRNA injection. Hence, the tests in the pest species will have to include oral feeding assays.

It had remained unclear whether plant protection efficiency would increase by expressing a dsRNA fragment targeting two target genes at the same time. Despite extensive testing we did find no a synergism between our most effective RNAi target genes.

Further, our analysis indicates that the number of off target sites does not strongly correlate with protein conservation and that it will be difficult if not impossible to design dsRNA fragments that do not have off target effects in any non-target species. One conclusion is that the search for RNAi target genes does not need to be restricted to species or taxon specific genes. The second conclusion is that the design of the target sequences needs to focus on avoiding off target effects on selected species that should be protected (like for instance bees) or that are most common in the respective environment.

## Methods

### Strains

*San Bernadino* strain (*SB*) was used and reared on wholegrain flour supplemented with 5 % yeast powder at 32 °C for all experiments.

### RNAi

All dsRNAs were ordered from Eupheria Biotech GmbH (www.eupheria.com), titrated from 0,5 ng/μl to 1 μg/μl and approximately 0.15 μl were injected into 10 larvae or adult beetles, respectively, using established techniques [30]. For the best 40 target genes non-overlapping fragments (1 μg/μl) were injected in additional RNAi experiments as a control for off-target effects. RNAi targeting the pigmentation gene *Tc-ebony* was used as negative control in all experiments at the respective concentrations. The change in body color confirmed successful RNAi while the survival rate was documented as negative control of the experiment. In the iBeetle screen 251 buffer injections were added as buffer control with 97 % not displaying any phenotype [24]. The survival rate was scored every second day post treatment by counting those animals that still showed movements. This number was divided by the number of injected animals in order to calculate the percentage of survival. All primers and sequences are documented at iBeetle Base [23].

### GO term clustering and identification of novel potential RNAi target genes

DAVID 6.7 (The Database for Annotation, Visualization and Integrated Discovery) [28, 29] was used to analyse the 11 and 40 RNAi most efficient target genes. The *Drosophila* genome was set as background and *p*-values (*p*-value ≤ 0.05) represented a modified Fisher’s exact *t*-test. The enriched GO terms were clustered into classes by the functional annotation clustering tool, which uses a grouping algorithm based on the hypothesis that similar genes should share similar annotations. The option settings were: classification stringency “high” and enrichment thresholds “EASE 0.05”. The enrichment score of each group is the geometric mean (in-log) of the *p*-values in an annotation cluster. Thus, the uppermost group shows the highest biological significance. GO Fat category was used for this analysis, which is a subset comprising more specific terms. The clustering of the eleven RNAi target genes was done with ten genes in total, because L67 was not associated with any GO term. For the top 40 clustering, 37 genes were used, due to three genes without associated GO terms. Note that the last DAVID GO database update was in 2009, some GO terms have changed in the last years and could be wrongly annotated in the clusters. In order to find further potential RNAi target genes, the GO terms of each cluster were used in the Flybase Query Builder [27]. With the obtained Flybase gene IDs we searched for *Tribolium* orthologs in the iBeetle Base [23]. Genes that caused a mortality of ≥70 % in the screen on day 11 after pupal or larval injection were assumed to be novel potential RNAi target genes.

### Off target analysis

The nucleotide sequence of the RNAi target genes (Query) was used to identify potential off target sites in transcript sequences of other species by Blast analysis. To this end, the length of the exact match was defined as ≥15 nt by the word size function at NCBI Blast and the match/mismatch score was defined as 1/−4. Exact matches smaller than 17 nt were excluded manually.

### Ethics statement

All experiments have been performed with invertebrate animals according to the respective institutional, national, and international guidelines (Directive 2010/63/EU). Approval by an ethics committee was not required.

## Additional files

Additional file 1: Table S1. lists the top 100 RNAi target genes with their *Tribolium* iBeetle-numbers, *Tc*-genes and *Drosophila* orthologs. **Table S2.** lists the best 40 RNAi target genes with their *Tribolium* iBeetle-numbers, *Tc*-genes and *Drosophila* orthologs. **Table S3.** lists the number of surviving animals after injection with non-overlapping dsRNA fragments. **Table S4.** lists the number of surviving larvae after single and double RNAi treatments (total dsRNA concentration 0,5 ng/μl). **Table S5.** shows the sequences of the *Tribolium* orthologs of the RNAi target genes published by Baum et al. [15]. (XLSX 37 kb) Additional file 2: Figure S1. shows the results for the most efficient 40 RNAi target genes*.* In **Figure S2.** the results of dsRNA injection into adult beetles are displayed. **Figure S3.** shows the phylogenetic trees of the novel RNAi target genes. **Figure S4.** shows the GO term clusters of the top 40 RNAi target genes. (PDF 3696 kb)
