# Supplementary material for: Large scale RNAi screen in Tribolium reveals novel target genes for pest control and the proteasome as prime target
Source: BMC Genomics. 2015 Sep 3;16(1):674. doi: 10.1186/s12864-015-1880-y (PMC4559001; doi:10.1186/s12864-015-1880-y)
Supplement: Additional file 2: Figure S1. — shows the results for the most efficient 40 RNAi target genes. In Figure S2. the results of dsRNA injection into adult beetles are displayed. Figure S3. shows the phylogenetic trees of the novel RNAi target genes. Figure S4. shows the GO term clusters of the top 40 RNAi target genes. (PDF 3696 kb) [file 12864_2015_1880_MOESM2_ESM.pdf]

Additional File 2

Figure S1: Identification of forty novel RNAi target genes in *Tribolium castaneum*

A

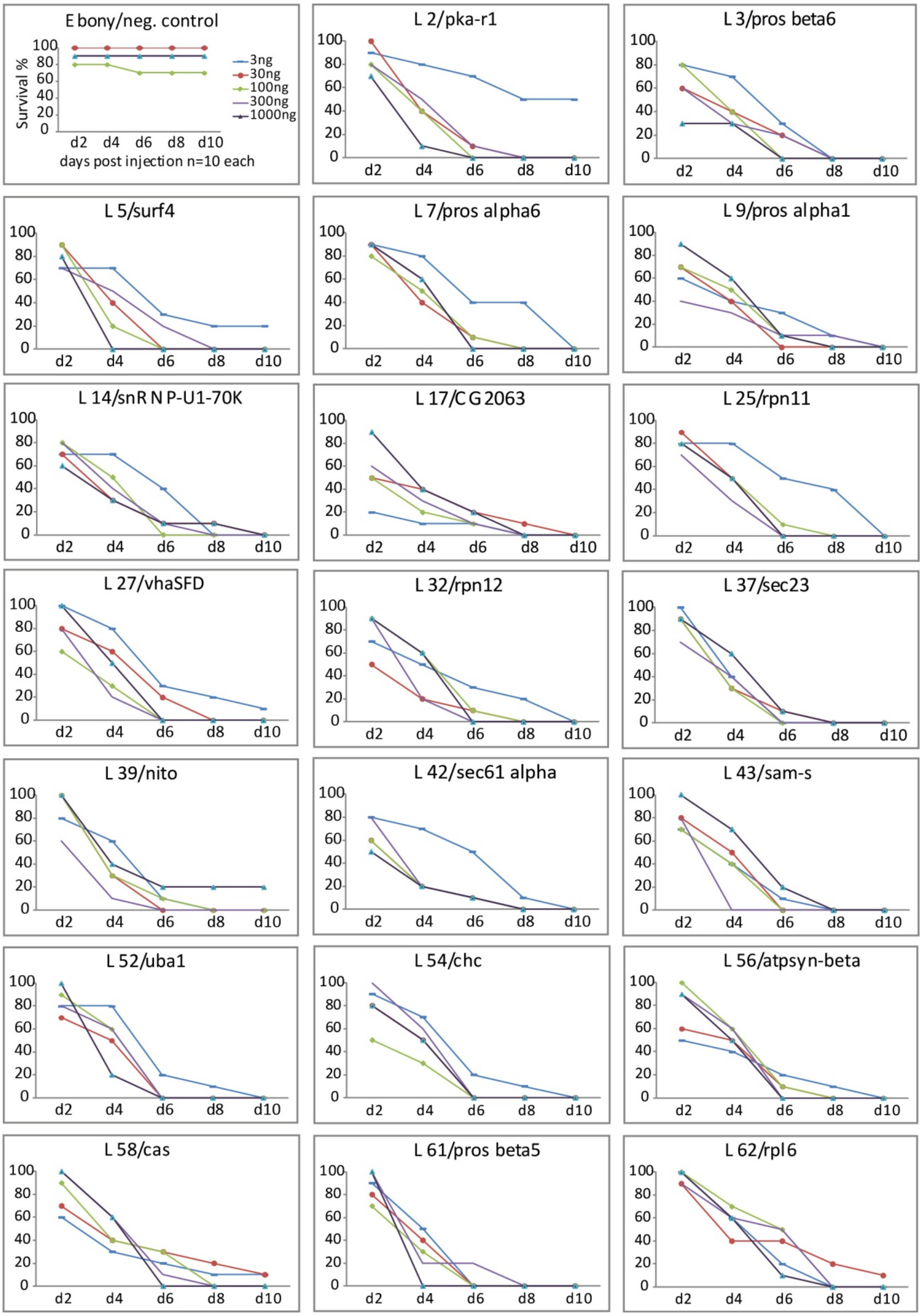

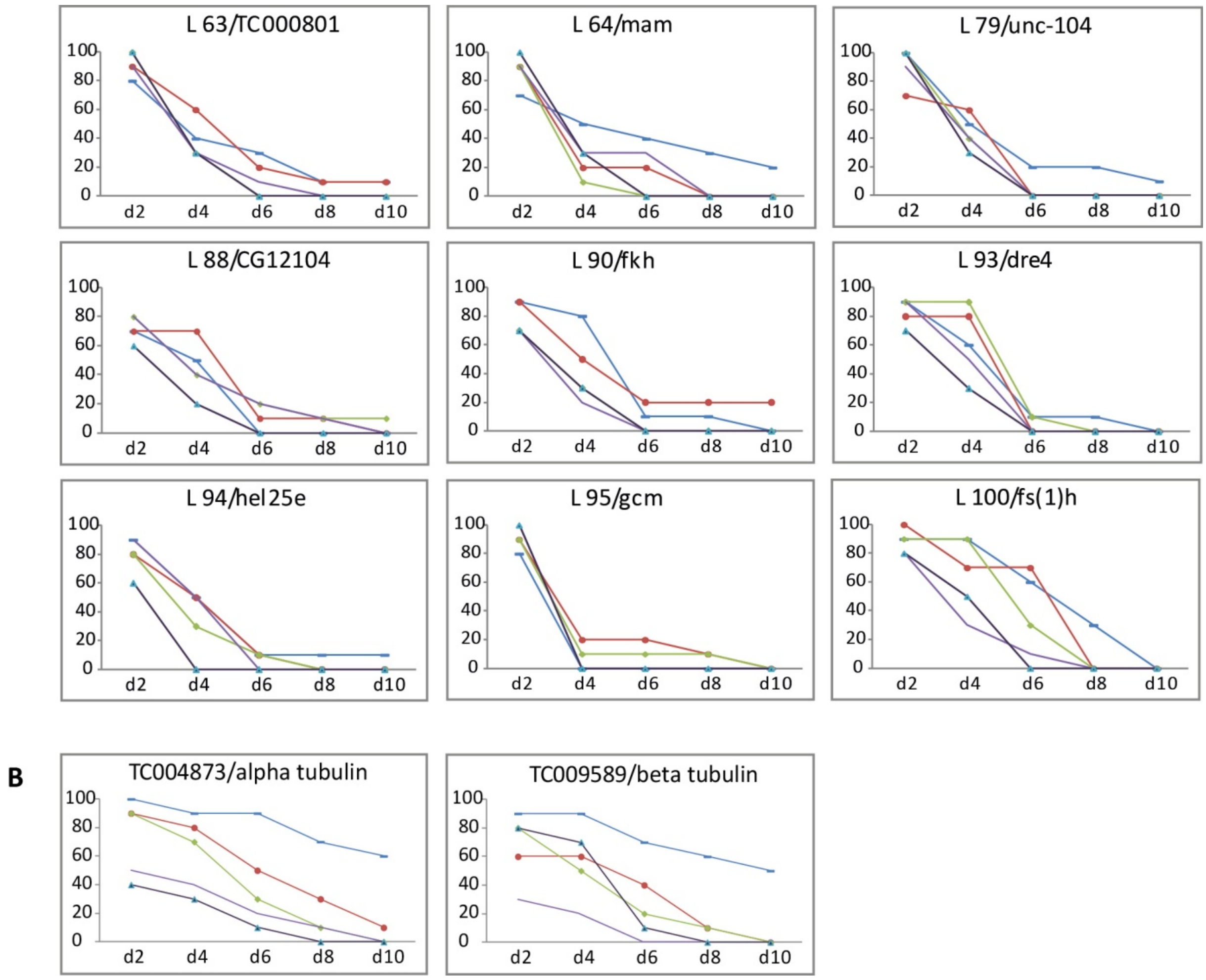

Based on data of the iBeetle screen, the 100 most efficient RNAi target genes were selected and retested using different dsRNA concentrations. (A) The results for the most efficient 40 lethal genes are shown. The corresponding results of the top eleven candidates are shown in Figure 1. See further details in figure legend of Figure 1. (B) The *Tribolium* orthologs of the two less effective RNAi target genes published by Baum et al., 2007 [1] are displayed.

Figure S2: Lethality is induced in adult stages as well

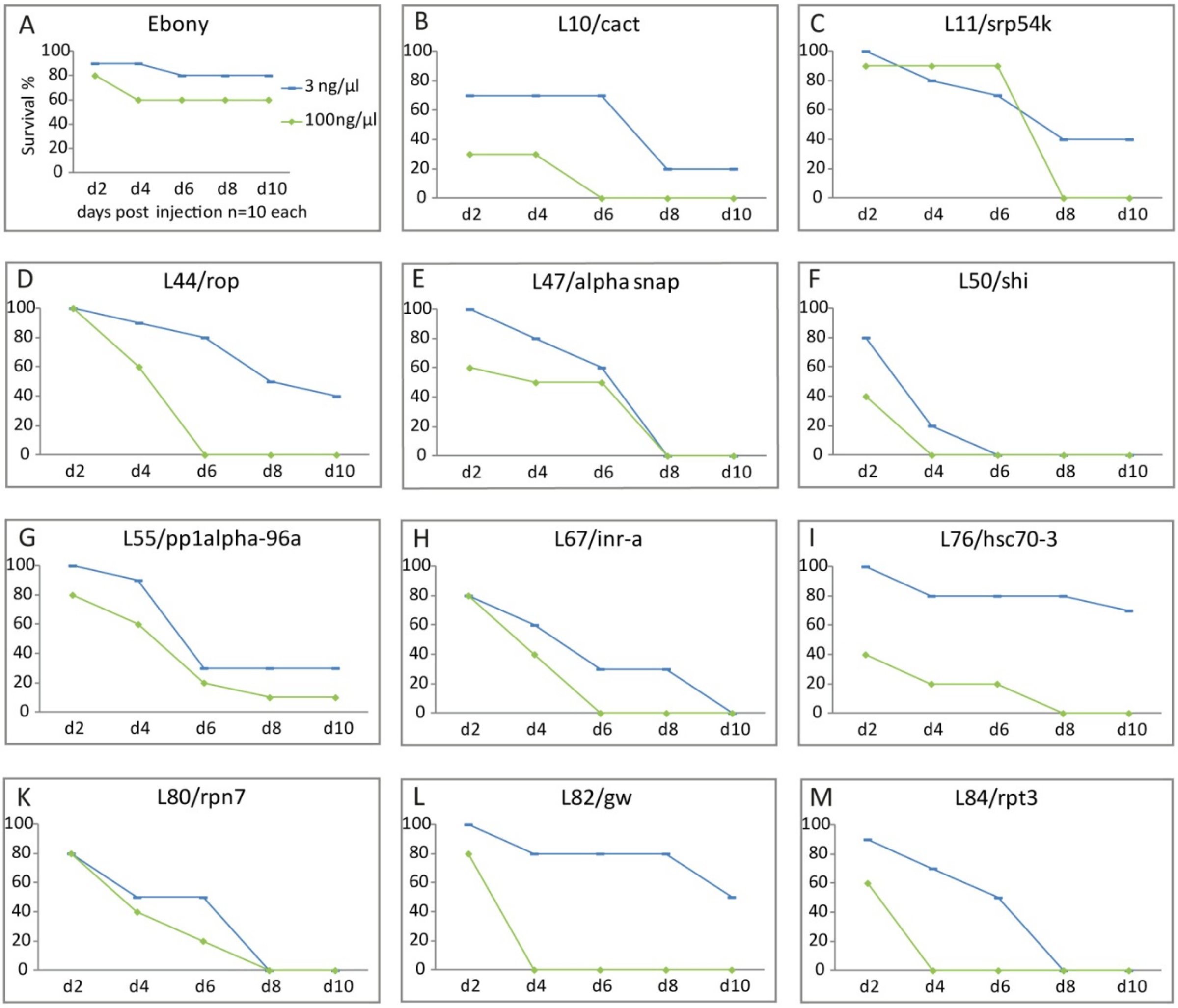

The most efficient eleven RNAi target genes were also tested at two different concentrations (3ng/µl, 100ng/µl) by injections into adult beetles. (A) *Tc-ebony* was used as negative control. (B-M) All RNAi target genes are lethal in adult stages as well but at lower concentrations, the efficiency was slightly lower compared to larval injections shown in Figure 1.

Figure S3: Phylogenetic trees of the novel RNAi target genes

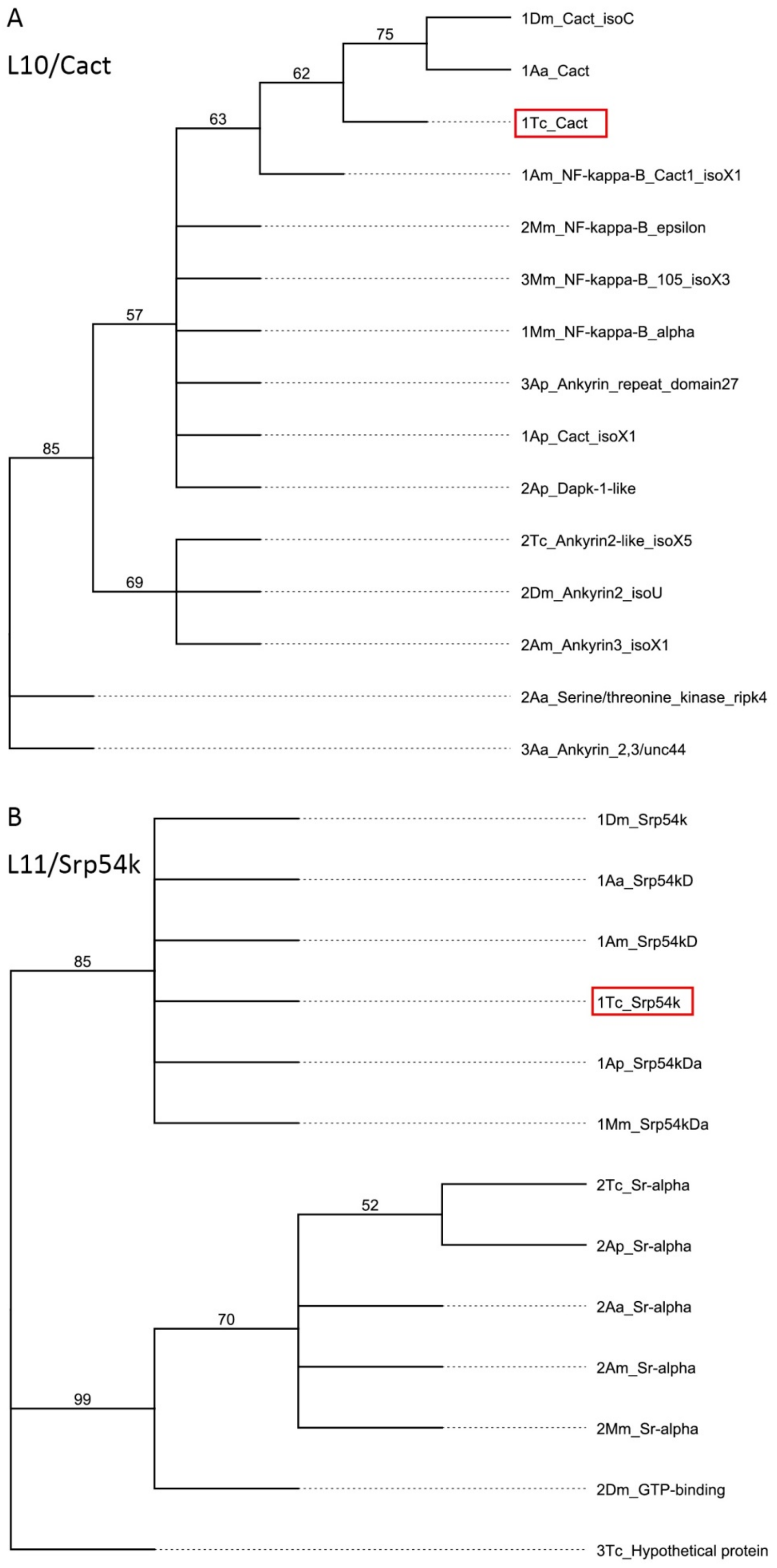

C

L44/Rop

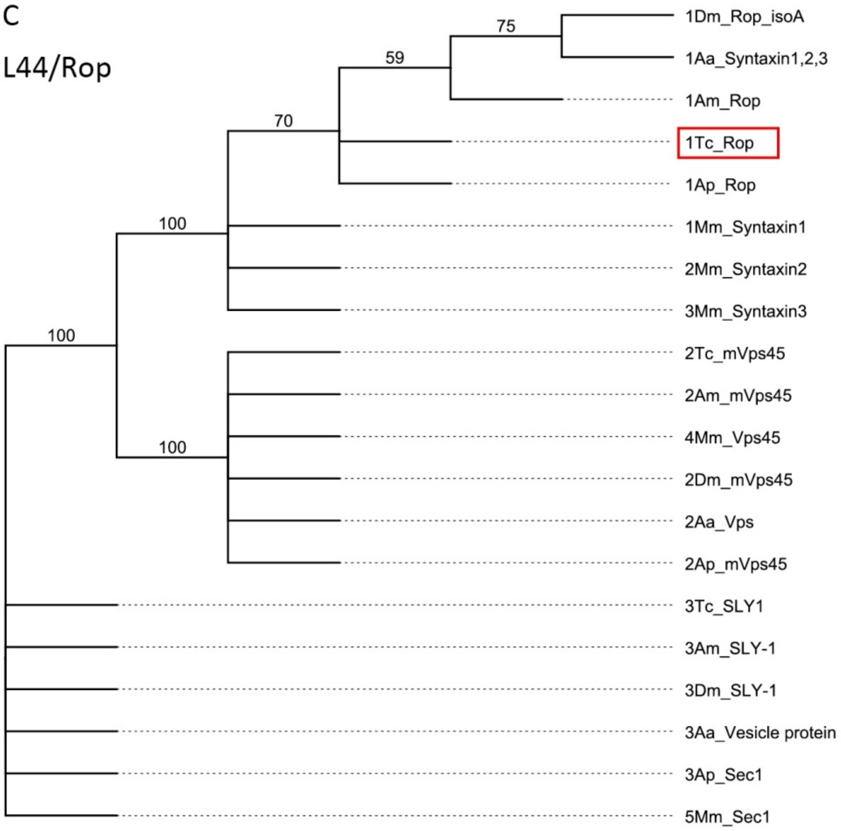

D

L47/Snap

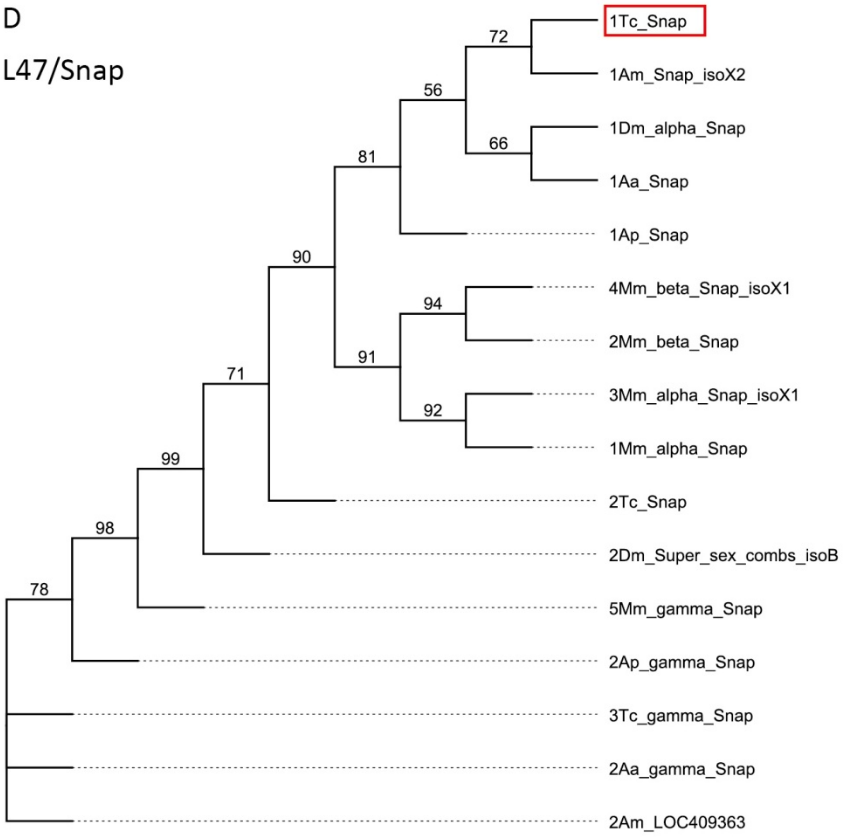

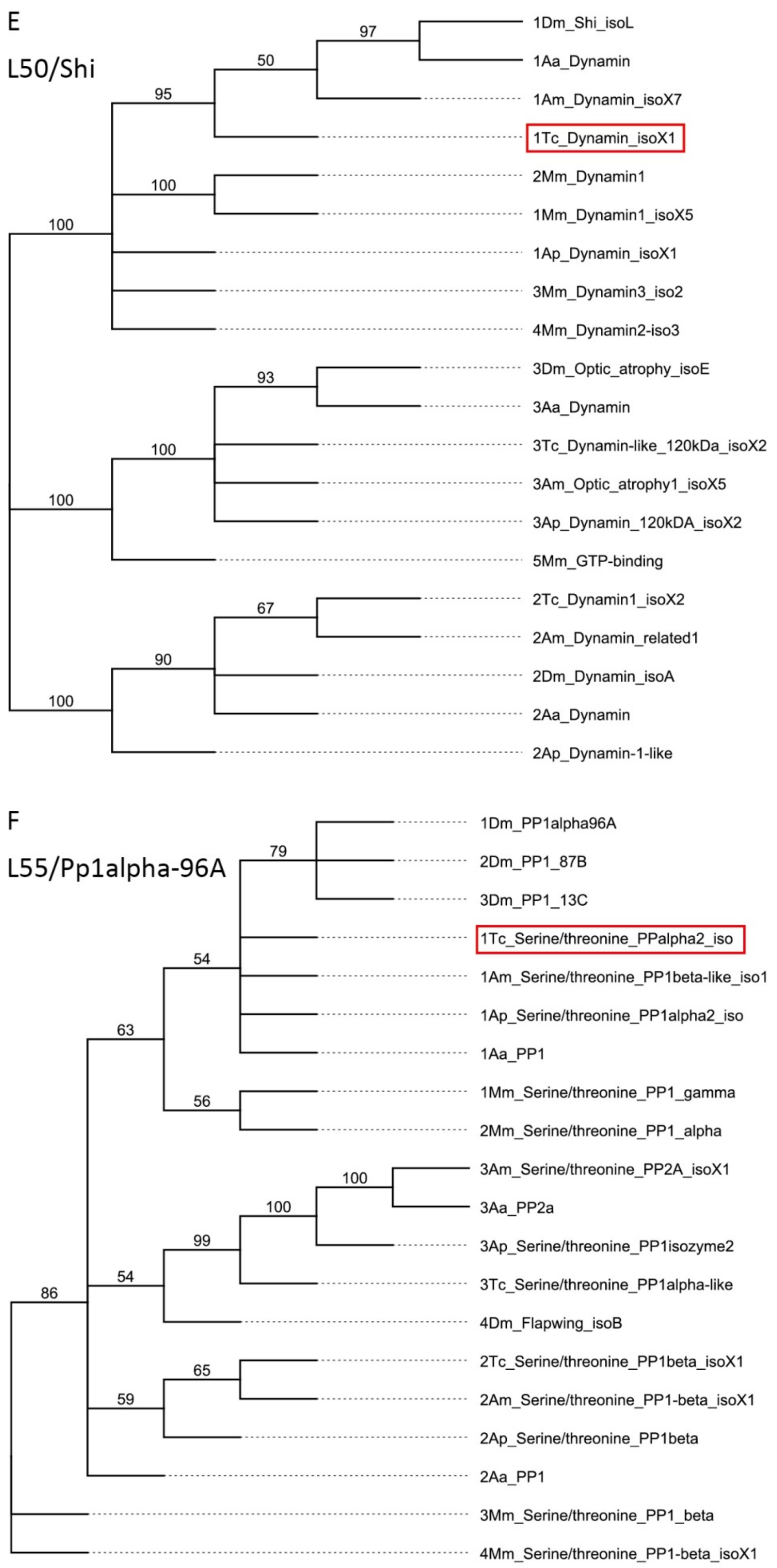
E
L50/Shi
97
50
95
100
100
93
100
100
67
90
100
1Dm_Shi_isoL
1Aa_Dynamin
1Am_Dynamin_isoX7
1Tc_Dynamin_isoX1
2Mm_Dynamin1
1Mm_Dynamin1_isoX5
1Ap_Dynamin_isoX1
3Mm_Dynamin3_iso2
4Mm_Dynamin2-iso3
3Dm_Optic_atrophy_isoE
3Aa_Dynamin
3Tc_Dynamin-like_120kDa_isoX2
3Am_Optic_atrophy1_isoX5
3Ap_Dynamin_120kDA_isoX2
5Mm_GTP-binding
2Tc_Dynamin1_isoX2
2Am_Dynamin_related1
2Dm_Dynamin_isoA
2Aa_Dynamin
2Ap_Dynamin-1-like
F
L55/Pp1alpha-96A
79
54
63
56
100
100
99
54
86
65
59
1Dm_PP1alpha96A
2Dm_PP1_87B
3Dm_PP1_13C
1Tc_Serine/threonine_PPalpha2_iso
1Am_Serine/threonine_PP1beta-like_iso1
1Ap_Serine/threonine_PP1alpha2_iso
1Aa_PP1
1Mm_Serine/threonine_PP1_gamma
2Mm_Serine/threonine_PP1_alpha
3Am_Serine/threonine_PP2A_isoX1
3Aa_PP2a
3Ap_Serine/threonine_PP1isozyme2
3Tc_Serine/threonine_PP1alpha-like
4Dm_Flapwing_isoB
2Tc_Serine/threonine_PP1beta_isoX1
2Am_Serine/threonine_PP1-beta_isoX1
2Ap_Serine/threonine_PP1beta
2Aa_PP1
3Mm_Serine/threonine_PP1_beta
4Mm_Serine/threonine_PP1-beta_isoX1

G

L67/In r-a

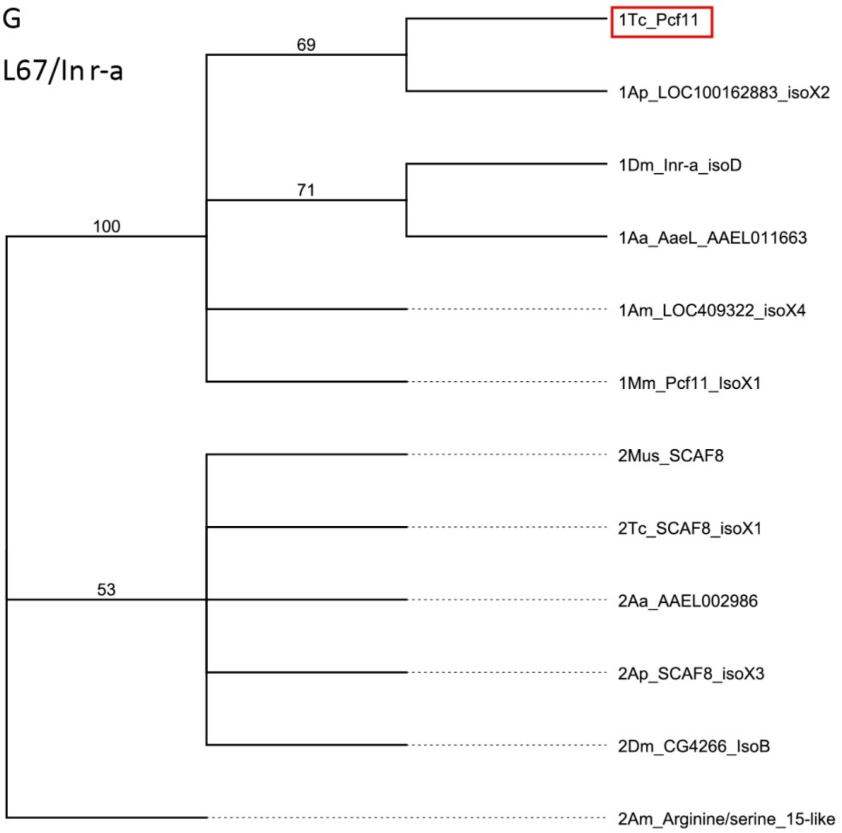

H

L76/Hsc70-3

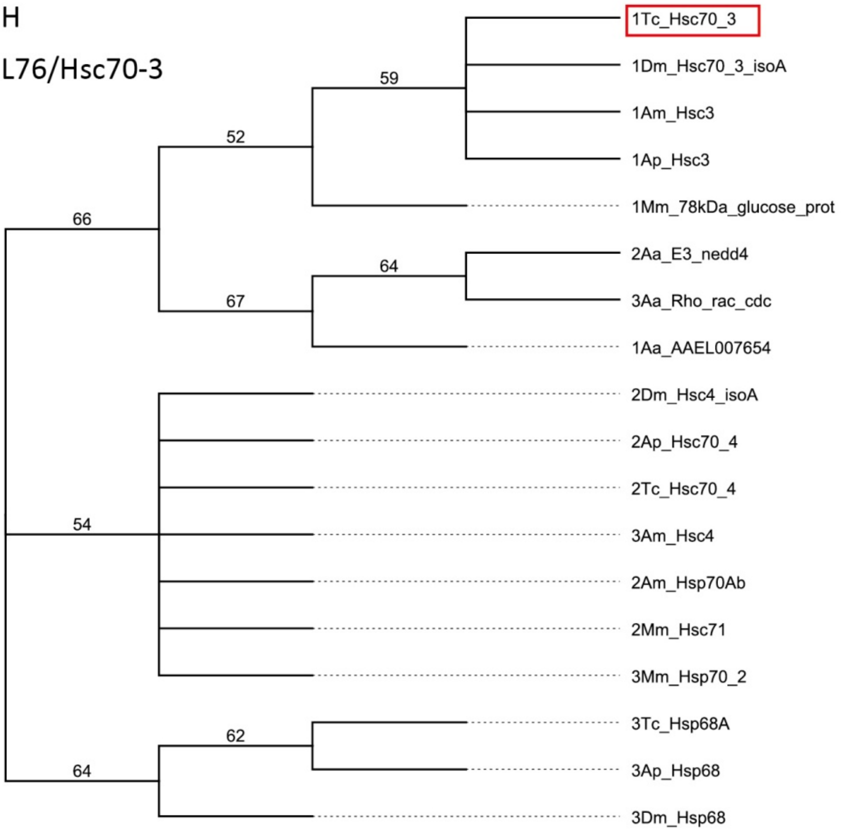

I

L80/Rpn7

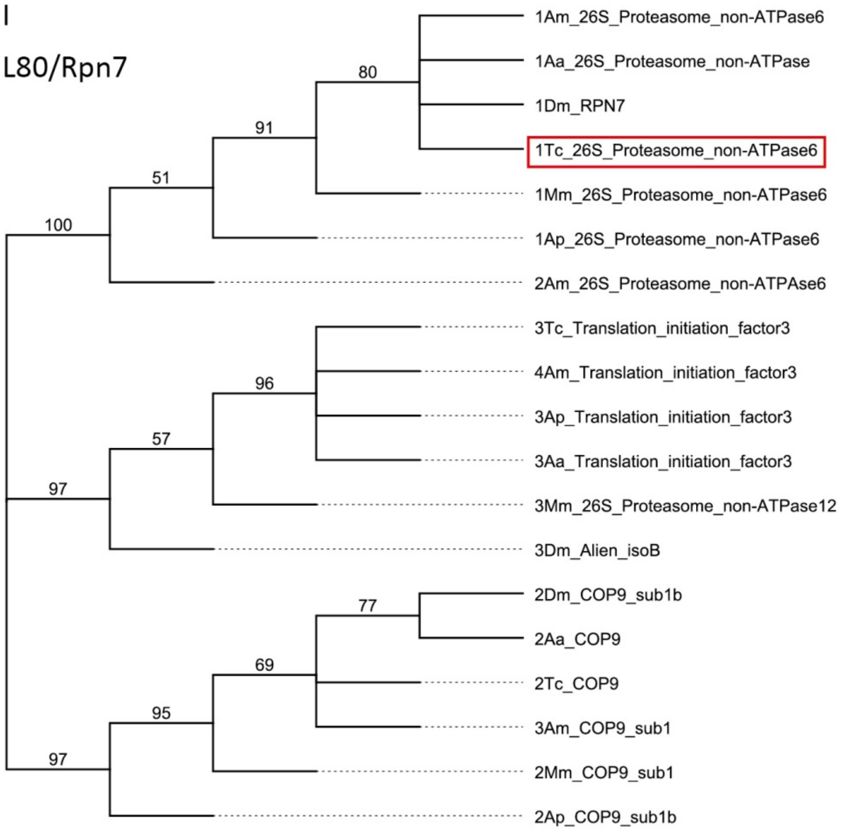

K

L82/Gw

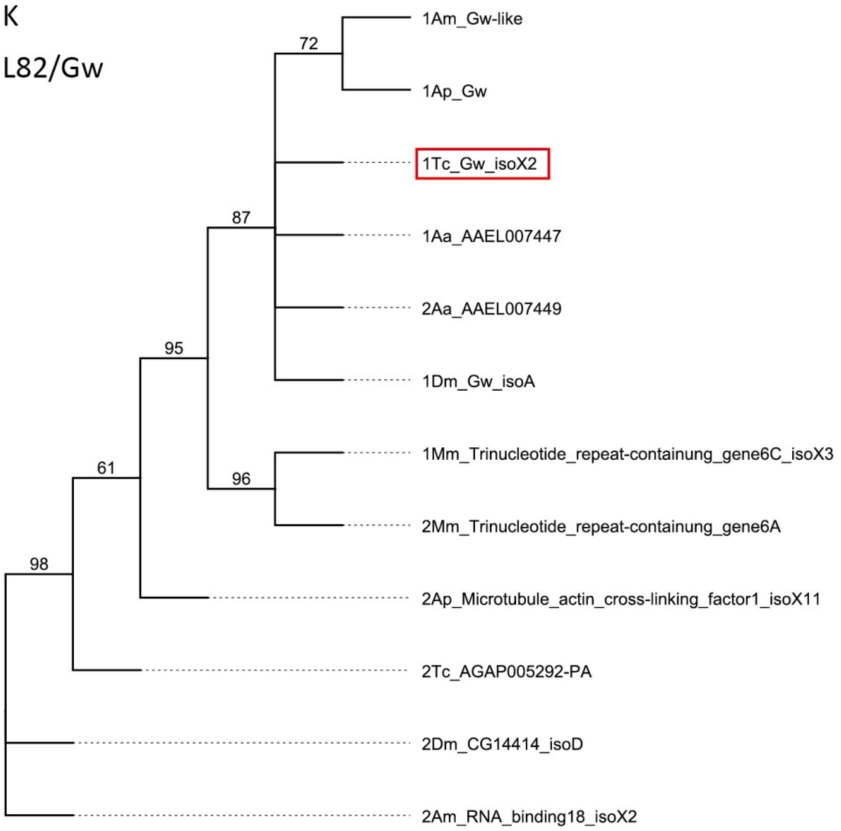

L

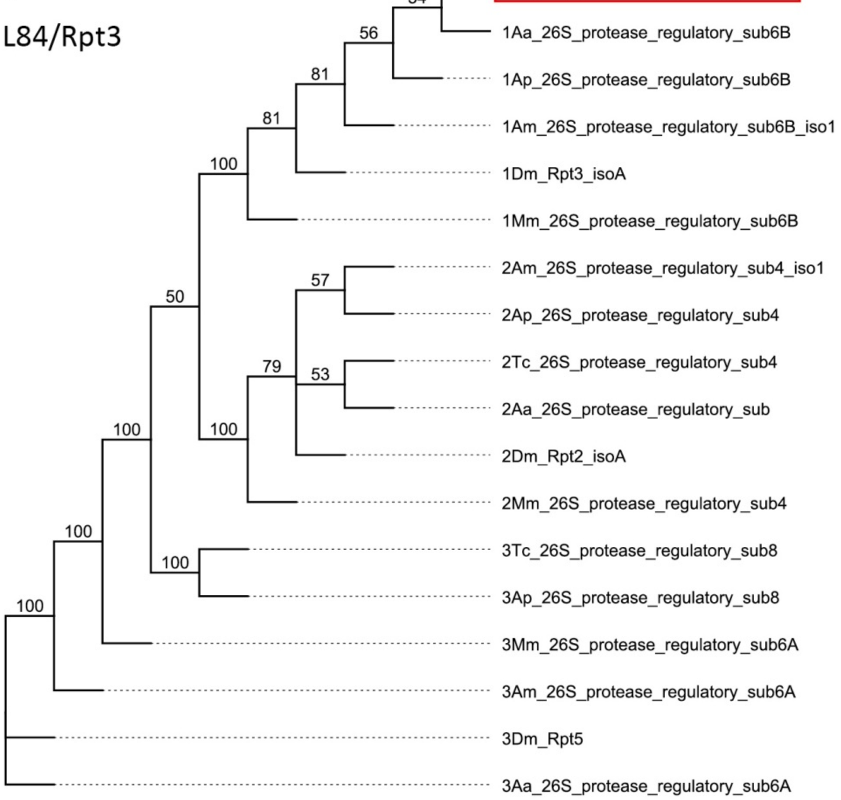

M

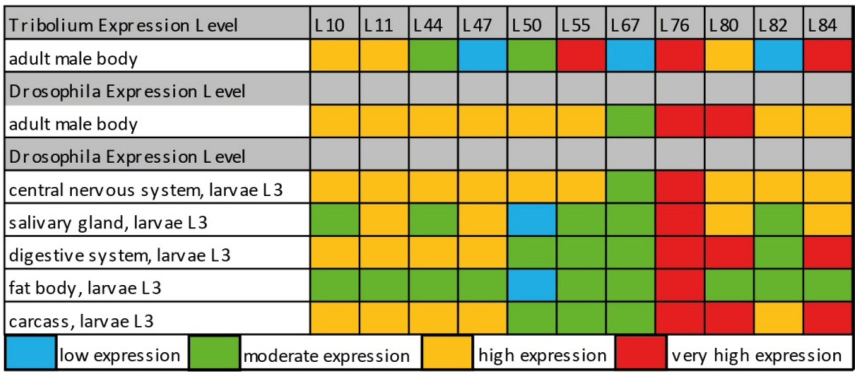

(A-L) The *Tribolium* protein sequences were blasted against *Drosophila melanogaster*, *Apis mellifera*, *Aedes aegypti*, *Acyrthosiphon pisum* and *Mus musculus* RefSeq protein collection [2] at NCBI (http://blast.ncbi.nlm.nih.gov/Blast.cgi). The alignments were done using ClustalW as implemented in the Geneious program (v.5.6.4) (Biomatters, Auckland, New Zealand) and trimmed to remove unclear parts of the alignments. The phylogenetic trees were calculated using the Geneious Tree Builder with the Jukes Cantor genetic distance model, neighbor-joining [3] as tree building method, and a number of 10.000 replicates for

creation of the bootstrap consensus tree [4]. Dm *Drosophila melanogaster*, Am *Apis mellifera*, Aa *Aedes aegypti*, Ap *Acyrthosiphon pisum,* Mm *Mus musculus*.

(M) Expression levels of the eleven RNAi target genes of *Tribolium* adult male body were obtained from RNA-Seq data of the *Tribolium* au2 gene set (http://bioinf.uni-greifswald.de/tcas/genes/au2/) and compared to the expression levels of the orthologous *Drosophila* genes in the adult male body obtained from modENCODE high-throughput RNA-Seq data in Flybase [5]. *Tribolium* expression levels were calculated and categorized into four expression strengths: low expression with 0-1.5, moderate expression with 1.6-3, high expression with 3.1-4.5 and very high expression with ≥4.6 number of reads per position. The comparison revealed similar expression levels only for L10, L11 and L76 in the adult male body of *Tribolium* and *Drosophila*.

Figure S4: GO term analysis of top 40 RNAi target genes reveals additional targets

Annotation Cluster 1	Enrichment Score: 4,26			
Category	Term	Count	%	PValue
SP_PIR_KEYWORDS	proteasome	7	19	1,61E-09
GOTERM_CC_FAT	GO:0000502~proteasome complex	7	19	1,03E-06
KEGG_PATHWAY	dme03050:Proteasome	7	19	1,37E-06
GOTERM_MF_FAT	GO:0004175~endopeptidase activity	7	19	1,21E-02
GOTERM_BP_FAT	GO:0006508~proteolysis	8	22	2,39E-02
GOTERM_MF_FAT	GO:0070011~peptidase activity, acting on L-amino acid peptides	7	19	4,36E-02
Genes	CG16916, CG5378, CG18174, CG4157, CG4904, CG1782,CG4097, CG12323			
Annotation Cluster 2	Enrichment Score: 2,37			
SP_PIR_KEYWORDS	threonine protease	3	8	1,72E-03
INTERPRO	IPR001353:Proteasome, subunit alpha/beta	3	8	3,46E-03
GOTERM_MF_FAT	GO:0004298~threonine-type endopeptidase activity	3	8	5,05E-03
GOTERM_MF_FAT	GO:0070003~threonine-type peptidase activity	3	8	5,05E-03
GOTERM_CC_FAT	GO:0005839~proteasome core complex	3	8	9,76E-03
Genes	CG4904, CG4097, CG12323			
Annotation Cluster 3	Enrichment Score: 2,22			
GOTERM_BP_FAT	GO:0008104~protein localization	7	19	4,24E-03
GOTERM_BP_FAT	GO:0015031~protein transport	6	16	4,71E-03
GOTERM_BP_FAT	GO:0006886~intracellular protein transport	5	14	4,97E-03
GOTERM_BP_FAT	GO:0045184~establishment of protein localization	6	16	5,23E-03
GOTERM_BP_FAT	GO:0034613~cellular protein localization	5	14	5,49E-03
GOTERM_BP_FAT	GO:0070727~cellular macromolecule localization	5	14	1,59E-02
Genes	CG4659, CG15811, CG9539, CG6625, CG9012, CG5848, CG13281			
Annotation Cluster 4	Enrichment Score: 2,10			
GOTERM_BP_FAT	GO:0007268~synaptic transmission	5	14	6,65E-03
GOTERM_BP_FAT	GO:0019226~transmission of nerve impulse	5	14	7,55E-03
GOTERM_BP_FAT	GO:0007267~cell-cell signaling	5	14	9,74E-03
Genes	CG15811, CG18102, CG42341, CG6625, CG9012			
Annotation Cluster 5	Enrichment Score: 2,09			
GOTERM_BP_FAT	GO:0030163~protein catabolic process	6	16	1,12E-03
GOTERM_BP_FAT	GO:0044265~cellular macromolecule catabolic process	6	16	1,89E-03
GOTERM_BP_FAT	GO:0051603~proteolysis involved in cellular protein catabolic process	5	14	5,94E-03
GOTERM_BP_FAT	GO:0044257~cellular protein catabolic process	5	14	5,94E-03
GOTERM_BP_FAT	GO:0019941~modification-dependent protein catabolic process	4	11	2,99E-02
GOTERM_BP_FAT	GO:0043632~modification-dependent macromolecule catabolic process	4	11	3,04E-02
SP_PIR_KEYWORDS	Protease	4	11	3,37E-02
Genes	CG16916, CG18174, CG4904, CG1782, CG4097, CG12323, CG31992			
Annotation Cluster 6	Enrichment Score: 2,01			
SP_PIR_KEYWORDS	nucleotide-binding	9	24	6,56E-04
GOTERM_MF_FAT	GO:0032555~purine ribonucleotide binding	10	27	1,35E-02
GOTERM_MF_FAT	GO:0032553~ribonucleotide binding	10	27	1,35E-02
GOTERM_MF_FAT	GO:0017076~purine nucleotide binding	10	27	2,06E-02
GOTERM_MF_FAT	GO:0032559~adenyl ribonucleotide binding	8	22	3,71E-02
Genes	CG4659, CG16916, CG18102, CG4147, CG42341, CG11154, CG1782, CG7269, CG2674, CG8566			
Annotation Cluster 7	Enrichment Score: 1,92			
GOTERM_BP_FAT	GO:0000226~microtubule cytoskeleton organization	6	16	6,31E-03
GOTERM_BP_FAT	GO:0007052~mitotic spindle organization	5	14	7,28E-03
GOTERM_BP_FAT	GO:0007051~spindle organization	5	14	1,21E-02
GOTERM_BP_FAT	GO:0007010~cytoskeleton organization	6	16	3,67E-02
Genes	CG8749, CG42341, CG4157, CG12323, CG7269, CG11522			
Annotation Cluster 8	Enrichment Score: 1,66			
GOTERM_BP_FAT	GO:0043623~cellular protein complex assembly	4	11	4,40E-03
GOTERM_BP_FAT	GO:0034622~cellular macromolecular complex assembly	4	11	2,82E-02
GOTERM_BP_FAT	GO:0006461~protein complex assembly	4	11	2,90E-02
GOTERM_BP_FAT	GO:0070271~protein complex biogenesis	4	11	2,90E-02
GOTERM_BP_FAT	GO:0034621~cellular macromolecular complex subunit organization	4	11	4,75E-02
Genes	CG4659, CG15811, CG6625, CG13281			
Annotation Cluster 9	Enrichment Score: 1,62			
GOTERM_BP_FAT	GO:0010324~membrane invagination	5	14	1,92E-02
GOTERM_BP_FAT	GO:0006897~endocytosis	5	14	1,92E-02
GOTERM_BP_FAT	GO:0016044~membrane organization	5	14	3,80E-02
Genes	CG18102, CG6625, CG9012, CG5848, CG13281			
Annotation Cluster 10	Enrichment Score: 1,56			
GOTERM_BP_FAT	GO:0050657~nucleic acid transport	3	8	2,64E-02
GOTERM_BP_FAT	GO:0050658~RNA transport	3	8	2,64E-02
GOTERM_BP_FAT	GO:0051236~establishment of RNA localization	3	8	2,72E-02
GOTERM_BP_FAT	GO:0015931~nucleobase, nucleoside, nucleotide and nucleic acid transport	3	8	3,04E-02
Genes	CG17332, CG9012, CG7269			

The functional clusters as revealed by GO term analysis (DAVID database) using our top forty RNAi target genes are displayed. GOTERM_BP_FAT where BP means biological process; GOTERM_CC_FAT where CC means cellular component; GOTERM_MF_FAT where MF means molecular function; SP_PIR_KEYWORDS where PIR means protein information resource.

Supplementary References

1. Baum JA, Bogaert T, Clinton W, Heck GR, Feldmann P, Ilagan O, Johnson S, Plaetinck G, Munyikwa T, Pleau M, Vaughn T, Roberts J: **Control of coleopteran insect pests through RNA interference**. *Nat Biotechnol* 2007, **25**:1322–1326.

2. Altschul SF, Gish W, Miller W, Myers EW, Lipman DJ: **Basic local alignment search tool.** *J Mol Biol* 1990, **215**:403–410.

3. Saitou N, Nei M: **The neighbor-joining method: a new method for reconstructing phylogenetic trees**. *Mol Biol Evol* 1987, **4**:406–25.

4. Felsenstein J: **Confidence limits on phylogenies: An approach using the bootstrap**. *Evolution* 1985, **39**:783–791.

5. Graveley BR, Brooks AN, Carlson JW, Duff MO, Landolin JM, Yang L, Artieri CG, van Baren MJ, Boley N, Booth BW, Brown JB, Cherbas L, Davis CA, Dobin A, Li R, Lin W, Malone JH, Mattiuzzo NR, Miller D, Sturgill D, Tuch BB, Zaleski C, Zhang D, Blanchette M, Dudoit S, Eads B, Green RE, Hammonds A, Jiang L, Kapranov P, et al.: **The developmental transcriptome of Drosophila melanogaster**. *Nature* 2011, **471**:473–479.
